# Complete Genome Sequences of Four Novel *Lactococcus lactis* Phages Distantly Related to the Rare 1706 Phage Species

**DOI:** 10.1128/genomeA.00265-14

**Published:** 2014-07-10

**Authors:** Witold Kot, Horst Neve, Finn K. Vogensen, Knut J. Heller, Søren J. Sørensen, Lars H. Hansen

**Affiliations:** a Department of Biology, Faculty of Science, University of Copenhagen, Copenhagen, Denmark; b Department of Environmental Science, Aarhus University, Roskilde, Denmark; c Department of Microbiology and Biotechnology, Max Rubner-Institut, Kiel, Germany; d Department of Food Science, Faculty of Science, University of Copenhagen, Frederiksberg, Denmark

## Abstract

*Lactoccocus lactis* is a Gram-positive bacterium widely used in the dairy industry in the production of an array of cheeses and other fermented milk products. Here, we describe the sequencing and genome annotations of a set of four phages virulent to *L. lactis* and exhibiting similarities to phage 1706.

## GENOME ANNOUNCEMENT

Lactococcal phages are the cause of extensive problems in the dairy industry, resulting in significant losses during manufacturing processes (1–4). Currently, phages of *Lactococcus lactis* are divided into 10 genetically distant groups based on genomic and morphological traits (5, 6). Phage 1706, isolated from a French cheese sample in 1995, is one of the rare lactococcal siphophage types (7).

Lytic phages designated P078, P092, P118, and P162 were isolated from different raw milk samples in Germany throughout 1978. Since these phages revealed morphological similarities with a subgroup of well-known lactococcal P335 phages (i.e., BK5-T-like small isometric-headed *Siphoviridae* phages with approximately 240-nm-long flexible tails [5, 6]), they were reactivated from the freezer and studied in detail. Phage DNAs were extracted from phage lysates purified by CsCl gradient centrifugation, as described previously (8). The double-stranded DNA (dsDNA) genomes of phages P078, P092, P118, and P162 have lengths of 54,452 bp, 53,513 bp, 54,173 bp, and 55,584 bp, respectively. Their G+C% contents are 33.5% (phages P078, P092, and P118) and 33.3% (P162), which is slightly lower than that of 1706 (33.7%). All four phages have single-stranded 3′ protruding cohesive overhangs (5′-CGCCCCAGTC-3′), which is different from the sequence of 1706 (5′-GCCCTGTCT-3′).

DNA sequencing libraries were prepared using the Nextera XT DNA kit (Illumina, San Diego, CA), according to the manufacturer’s protocol. Individually tagged libraries were sequenced as a part of a flowcell as 2 × 250-base paired-end reads using the Illumina MiSeq platform (Illumina). The total read yield was between 614,624 (for P078) and 908,954 (for P162) reads per library. The reads were processed using CLC Genomics Workbench 6.0.4 (CLC bio, Aarhus, Denmark), as described previously (9). The average read lengths after trimming were 168 nucleotides (nt) (P078), 212 nt (P092), 154 nt (P118), and 215 nt (P162). The average coverages of the obtained contigs were in range of 1,746× (P078) to 3,439× (P162). Additionally, Sanger sequencing reactions were performed in order to determine the *cos*-site sequence, essentially as described before but using the custom primers cos_F (5′-CTCAATCAAGAAGCAAAGCTAA-3′) and cos_R (5′-AATGTTTAAGGAGAACACGTTG-3′) (8). The completed sequences were analyzed and functionally annotated using the RAST server (10). RAST analysis revealed 78, 76, 80, and 82 potential open reading frames (ORFs) for P078, P092, P118, and P162, respectively. All ORFs were predicted on the positive strand. The genomes of P078, P092, P118, and P162 share a high level of nucleotide similarity (89.4 to 93.6% for pairs P092-P162 and P078-P092, respectively). The majority of predicted ORFs are not assigned to any specific function. The closest relative of P078, P092, P118, and P162 seems to be phage 1706 (45% nucleotide identity). Local stretches of nucleotide similarities were short (max 1 kb) and limited (19 local similarities of length 500 to 1,000 bp, with nucleotide identity ranging from 25.8 to 93.3%). On the protein level, less than half of the predicted proteins (32/76 for P092) exhibit significant local similarities (*E* value, <10^-5^) to the translated proteins of phage 1706. Moreover, the genomes of the newly sequenced phages appear to be organized similarly to the 1706 genome.

All 4 phages were derived from raw milk samples, indicating that milk can be a source of new phage populations. The full-genome sequences of P078, P092, P118, and P162 provide new insight on phage evolution and biodiversity and can serve as a platform for the further characterization of industrially important lactococcal phages related to the 1706 species.

### Nucleotide sequence accession numbers.

Genome sequences have been deposited in GenBank under accession no. KJ489010 (P078), KJ489011 (P092), KJ489012 (P118), and KJ489013 (P162).
